# Inference of Gene Regulatory Networks for Breast Cancer Based on Genetic Modules

**DOI:** 10.34133/bmef.0154

**Published:** 2025-08-05

**Authors:** Yihao Chen, Ling Guo, Yue Pan, Hui Cai, Zhitong Bing

**Affiliations:** ^1^ College of Electrical Engineering, Northwest Minzu University, Lanzhou, China.; ^2^ School of Information Engineering, Yangzhou University, Yangzhou, China.; ^3^ Guangdong Provincial Key Laboratory of Malignant Tumor Epigenetics and Gene Regulation, Medical Research Center, Sun Yat-Sen Memorial Hospital, Sun Yat-Sen University, Guangzhou, China.; ^4^ Nanchang Research Institute, Sun Yat-Sen University, Nanchang, China.; ^5^ Gansu Province People Hospital, Lanzhou, China.; ^6^ Institute of Modern Physics, Chinese Academy of Sciences, Lanzhou, China.

## Abstract

**Objective:** Breast cancer is a common tumor and has a high mortality rate. Gene regulatory networks(GRNs) can genetically facilitate targeted therapies for this disease. **Impact Statement:** This study proposes a new method to infer GRNs. This new method combining genetic modules and convolutional neural networks is presented to infer GRNs from the RNA sequencing data of breast cancer. **Introduction:** GRNs play an essential role in many disease treatments. Previous studies showed that GRNs will accelerate tumor therapy. However, most of the existing network inference methods are based on large-scale gene collections, which ignore the characteristics of different tumors. **Methods:** In this work, weighted gene coexpression network analysis was deployed to screen key genes and gene modules. The gene regulatory associations in gene modules were then transformed into 2-dimensional histogram types. A convolutional neural network was chosen as the main framework to fit the gene regulatory types and infer the GRN. **Results:** The method integrates genetic data analysis and deep learning perspectives to screen key genes and predict GRNs among key genes. The key genes screened were validated by multiple methods, and the inferred gene regulatory associations were widely validated in real datasets. **Conclusion:** The method can be used as an auxiliary tool with the potential to predict key genes and the GRNs of key genes. It has the potential to facilitate the therapeutic process and targeted therapy for breast cancer.

## Introduction

Gene regulatory networks (GRNs) are complex bioinformatics models that describe the interactions between genes and transcription factors, nucleic acids, and proteins [1]. In GRNs, the expression of a single gene is directly or indirectly affected by other regulatory genes. It leads to a differential expression profile for the single gene in different cells [2]. The study of GRNs is important for understanding the principles of intracellular gene regulation as well as the pathogenetic mechanism and targeted therapies of diseases.

Over the past decades, a large number of computational models have been proposed to infer GRNs from biomics data, such as mutual information [3–5], matrix inference [6], probabilistic graphical models [7,8], Bayesian networks [9–11], Boolean networks [12–14], and difference analysis [15]. Mutual information is the information shared by 2 random variables or the reduction of uncertainty due to a known random variable [3]. Zhang et al. [4] used an algorithm of conditional mutual information to calculate dependency associations between genes from the covariance matrix of gene expression profiles for inferring GRNs. Since the execution result of the algorithm of conditional mutual information depends on the order of the input genes, the algorithm does not have high robustness. To address this issue, Aghdam et al. proposed a heuristic algorithm to find stable gene sequences. A reliability factor was also added to the edges in the GRNs, and the results showed that the method has high inference accuracy [5]. In addition, matrix inference methods have also been used to infer GRNs. Passemiers et al. [6] constructed accuracy matrices by linearly transforming the covariance matrix of the samples and the variance matrix of the gene expression to infer GRNs. Furthermore, probabilistic inference models are also considered suitable for inferring GRNs [7]. Using a randomized wandering and restart strategy, Liu et al. [8] proposed a global topology-based GRN inference method and achieved good results. Among probabilistic inference methods, Bayesian networks are also one of the common methods for inferring GRNs. In a Bayesian network, genes are mapped as nodes and edges are mapped as connection strengths between nodes [9]. Liu et al. proposed a new method based on local Bayesian networks and *K* nearest neighbors. This method solves the problem that mutual information cannot infer the type of gene regulation, but it is more time-consuming to select candidate genes [10]. To improve the efficiency of implementation, Xing et al. proposed a candidate auto-selection algorithm. It can automatically screen candidate genes based on mutual information and breakpoint detection [11]. In the GRN inference problem, researchers have also attempted to use discretized systems to infer GRNs such as Boolean networks [12]. Gamage et al. proposed a new feature selection method based on filters. This method uses a Boolean network with Pearson correlation coefficients to learn regulatory patterns among genes [13]. Li et al. developed the LogBTF method, which combines logistic regression and Boolean threshold networks to infer GRNs from discretized time-series data. The results showed that the method can infer GRNs from single-cell temporal expression data with high accuracy [14]. Guo et al. [15] used the overlap between 4 groups of differentially expressed genes to construct a regular network of 4 subtypes of breast cancer.

With the development of artificial intelligence, many methods of machine learning and deep learning have also been used to infer gene regulatory associations. Gillani et al. [16] developed the tool CompareSVM to infer GRNs, and it can measure gene-to-gene similarity based on kernel functions. With the rich information in protein–protein interaction (PPI) networks and transcription factor–DNA binding sites, Petralia et al. [17] proposed a random-forest-based inference method that integrates valid information from external data to accurately infer GRNs based on a sampling strategy. Yuan and Bar-Joseph [18] transformed gene associations into histograms and then used deep learning to fit association types between genes. Zheng et al. formulated a deep contrastive learning method to fit gene regulatory association from gene expression images during drosophila eye development. They compared and merged the losses of 2 independently trained convolutional neural networks (CNNs) to update the network parameters [19]. Gan et al. used a bidirectional recurrent neural network to infer GRNs from time-series single-cell RNA sequencing (RNA-seq)data. The excellent performance of the method on 4 simulated datasets and 3 real datasets demonstrates its ability to accurately infer GRNs [20].

Key genes play a vital role in tumor progression and anticancer medicine development [21]. Exploring these key genes and the GRNs among them helps to understand the influence of genes in tumor pathogenesis. Huang et al. [22] investigated the role of key genes in colorectal cancer development and metastasis and found that key genes contribute to targeted therapy. Some researchers have also tried to screen key genes from the perspective of GRNs. For example, Nazarieh et al. [23] proposed the minimum connected dominating set method to find key genes from the perspective of GRNs. However, it is worth noting that the above methods were implemented directly on a large amount of genetic data and did not filter the gene modules for the sample phenotype. Moreover, the key genes of different tumors were not screened to construct GRNs.

**Fig. 1.**
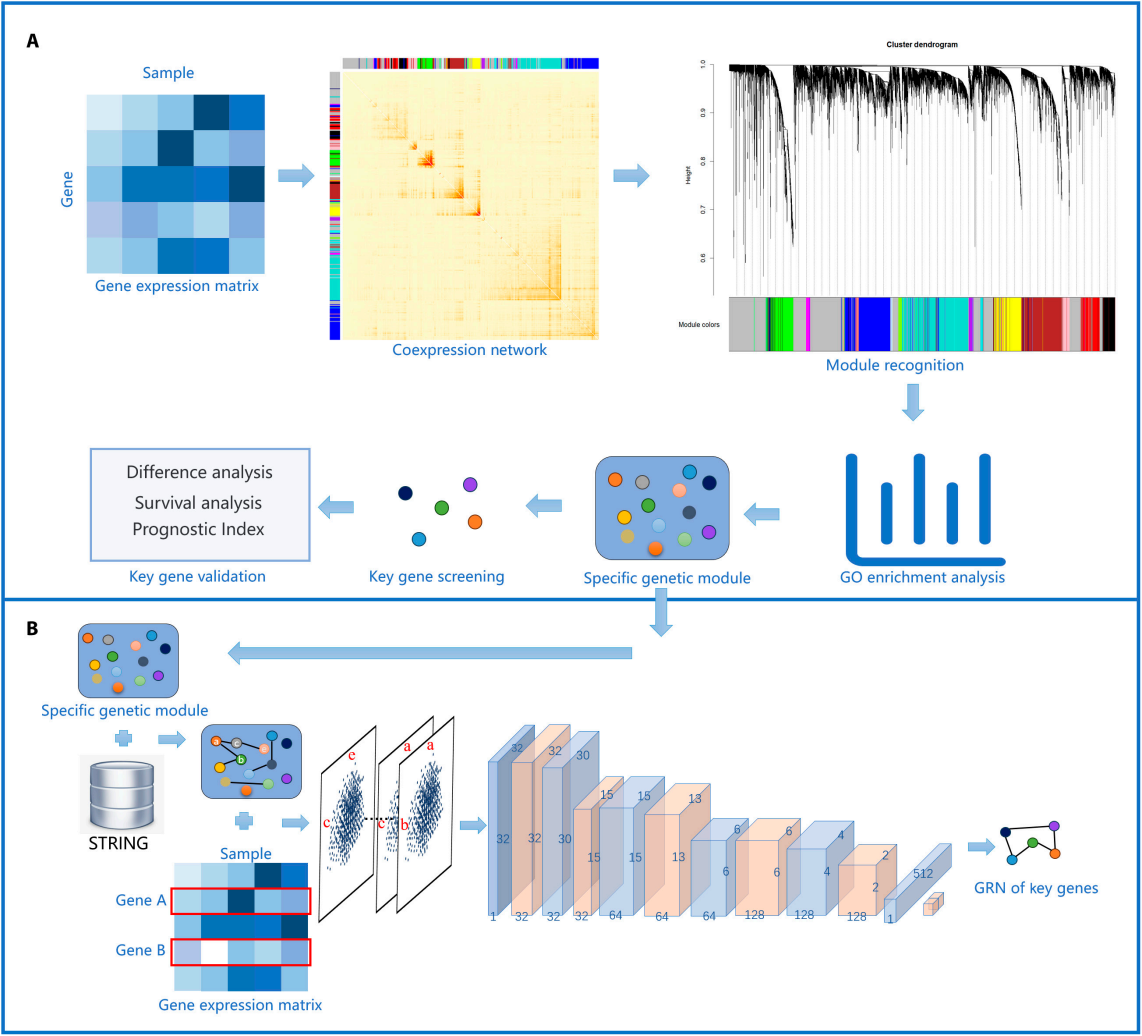
The framework of the method. (A) Screening of genetic modules and key genes: For gene expression data, construction coexpression networks to screen genetic modules and key genes. (B) Prediction of gene regulatory associations: Based on genetic modules and PPI networks, gene regulatory associations were transformed into 2-dimensional (2D) histograms. A convolutional neural network (CNN) was chosen as the main framework to fit gene regulatory associations by 2D histograms as input. Finally, the gene regulatory networks (GRNs) between key genes were predicted by this model. GO, Gene Ontology; STRING, Search Tool for Recurring Instances of Neighbouring Genes.

In our work, a new method combining genetic modules and CNNs is constructed to infer GRNs from RNA-seq data. Genetic modules are highly correlated collections of genes that represent stronger patterns of coexpression between genes. We focus on the relationship between gene coexpression networks and GRNs, and gene regulatory associations are investigated from the perspective of gene coexpression networks [15,24,25]. Weighted gene coexpression network analysis (WGCNA) was employed to screen genetic modules and reveal the key genes in breast cancer. Next, we constructed a model using CNNs and a PPI database to predict the GRNs for the key genes in breast cancer. The method consists of 2 steps, and its framework is shown in Fig. 1: (a) Screening of genetic modules and key genes: WGCNA is employed to identify genetic modules, and Gene Ontology (GO) enrichment analysis is used to screen key genetic modules. These gene modules are significantly associated with tumor. Meanwhile, the key genes in the key genetic modules are screened and validated by various methods. (b) Prediction of gene regulatory associations: The PPI network in the key genetic module was chosen as a prior network. In the prior network, if there is a regulatory association between 2 genes, it is considered a positive sample; otherwise, it is a negative sample. It is worth noting that all positive and negative samples will be transformed into histogram types. Finally, a CNN was chosen as the main framework of the method to fit the regulatory types. The method was also executed on multiple datasets and compared with multiple GRN methods. Two public databases validated the accuracy of the method for inferring GRNs. The rest of the paper is organized as follows: Materials and Methods describes the method in 2 main steps, Results shows the results of the method on the dataset and a comparison of the method with other common GRN methods, and Conclusion briefly concludes this work.

## Results

### Genetic modularity

#### Selection of a reasonable soft threshold

**Fig. 2.**
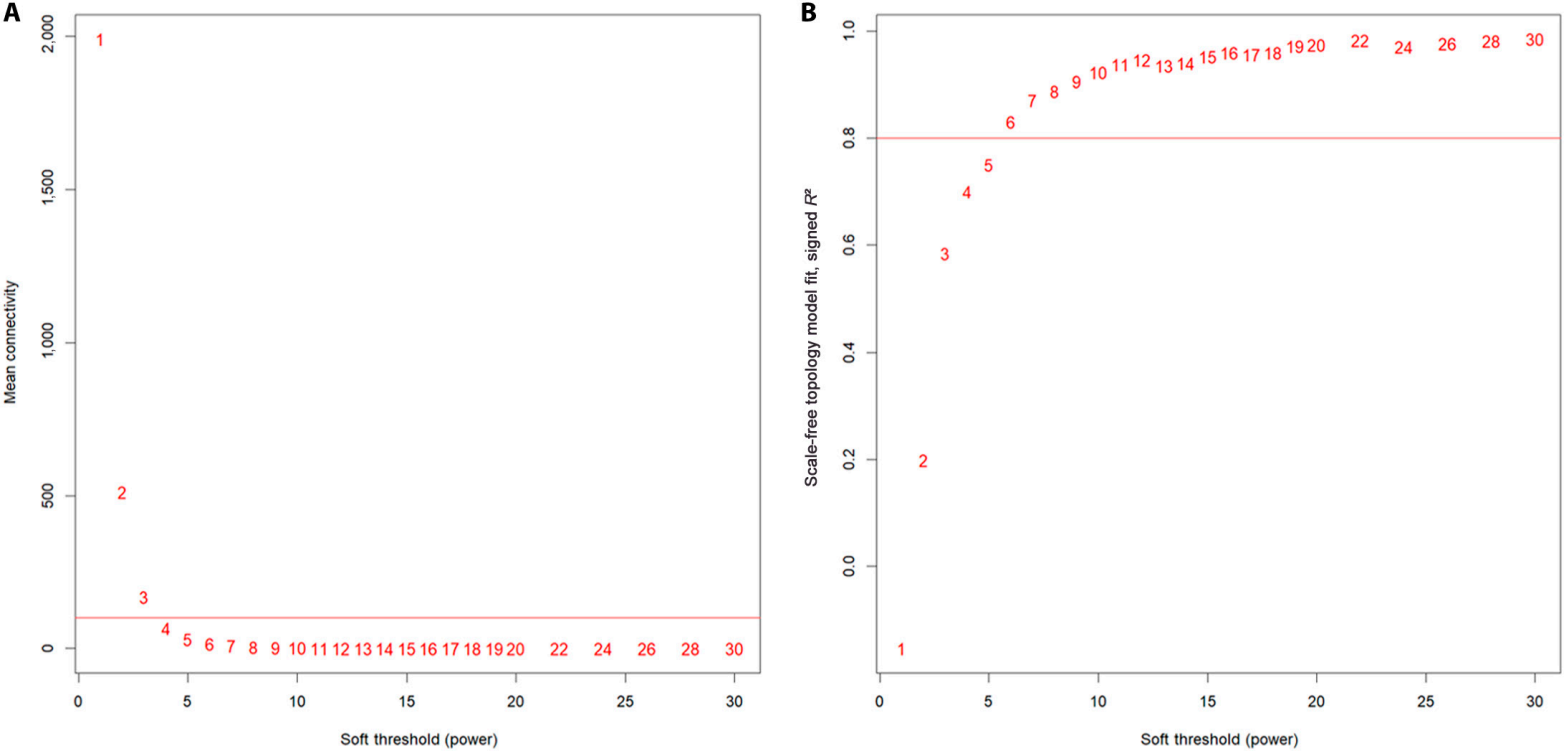
Soft threshold selection. (A) The average connectivity of the network under different soft thresholds. In scale-free networks, the average network connectivity is generally small. (B) Scale-free fitting indices under different soft thresholds. The red line indicates the chosen value of the scale-free fitting index, which is set to 0.8, and is generally chosen to be between 0.8 and 0.9.

The work of WGCNA is based on 2 assumptions: (a) genes with similar expression patterns may be coregulated, functionally related, or in the same pathway. (b) The gene network conforms to a scale-free distribution. An important process in constructing WGCNA networks is the selection of a soft threshold. Suitable soft thresholds are more adaptable to real biological processes, continuously adapt to the input data by weighted summation, and maximize gene correlation networks to conform to scale-free networks. In this article, we extracted breast cancer gene expression data from The Cancer Genome Atlas (TCGA) database to study. We separately evaluated the scale-free net compliance for soft thresholds taken from 1 to 30. The soft threshold selection is shown in Fig. 2. Figure 2 exhibits the degree of approximation of the initial network with respect to the scale-free network for different values of soft threshold β.

When signed $R^{2}>0.85$, the minimum power β is equal to 6 and the topology of the weighted gene coexpression network is highly consistent with the scale-free network. Therefore, the final value of parameter β is chosen as 6.

#### Module identification in WGCNA

**Fig. 3.**
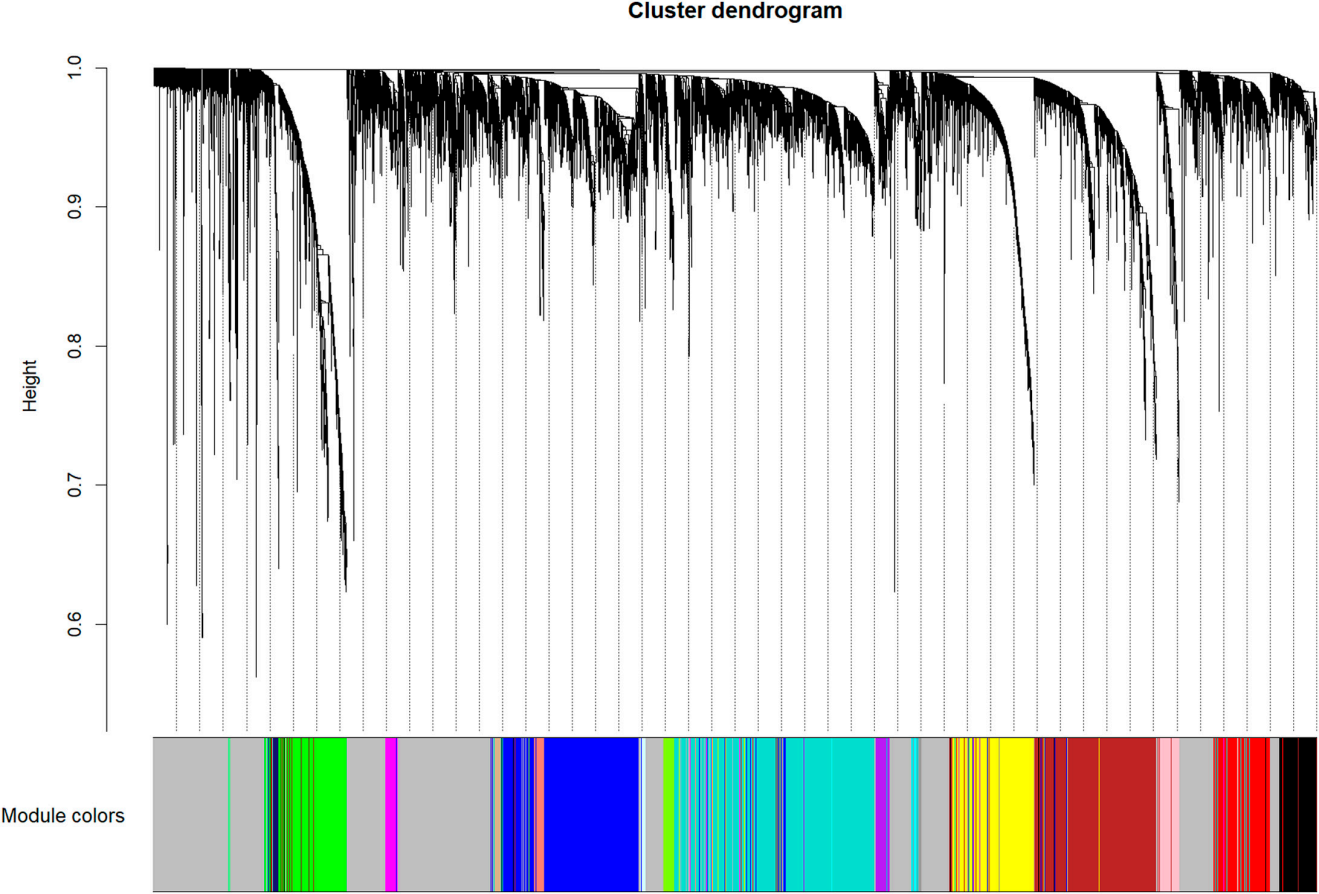
Coexpression network dendrogram.

After the soft threshold parameter was determined, all possible associations between 15,000 genes were calculated in parallel and recorded in the correlation matrix A. Coexpression linkage strengths between genes were stored in the corresponding entries in matrix A and were represented by the range 0 to 1. Higher values indicate stronger coexpression between genes, while lower values indicate a weaker correlation. WGCNA networks are constructed to emphasize high correlation at the expense of low correlation by performing power operations with a soft threshold, which makes the correlation values more biologically meaningful in line with scale-free network characteristics. Hierarchical clustering was applied to obtain highly inline sets of genes from the correlation matrix, called genetic modules. The first principal component gene of the module was chosen as the main gene, called module eigengene, which represents the gene expression profile of the whole module. After weighted gene coexpression networks of breast cancer were constructed, 18 modules were obtained by hierarchical clustering. Because a gray module represents unidentifiable genes in WGCNA, the gray module was eliminated in our study. The coexpression network dendrogram is displayed in Fig. 3. In the present study, the minimum size of each module is 30. It means that each module contains at least 30 genes.

#### Filtering of modules

**Fig. 4.**
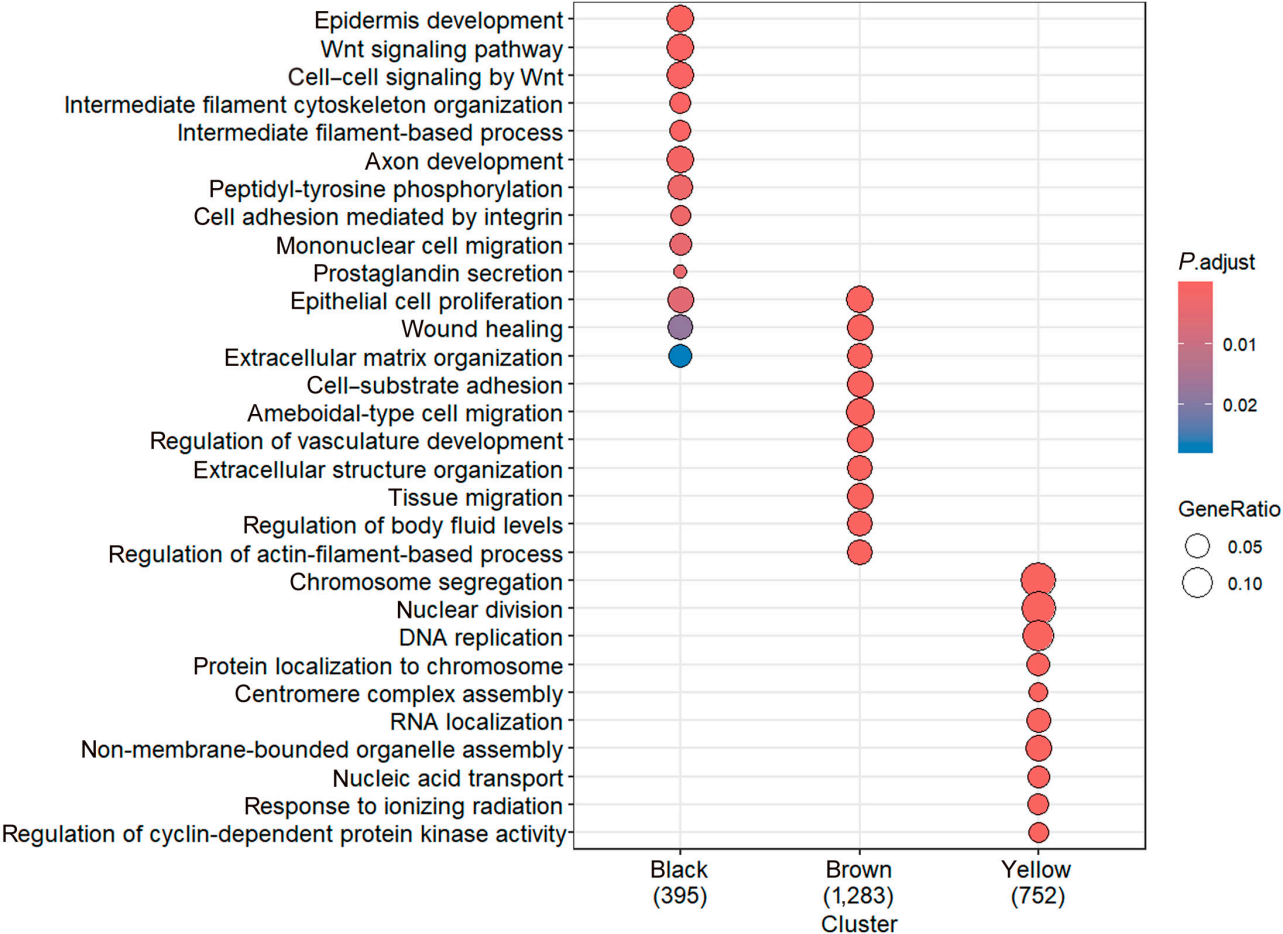
GO enrichment pathway of modules.

**Fig. 5.**
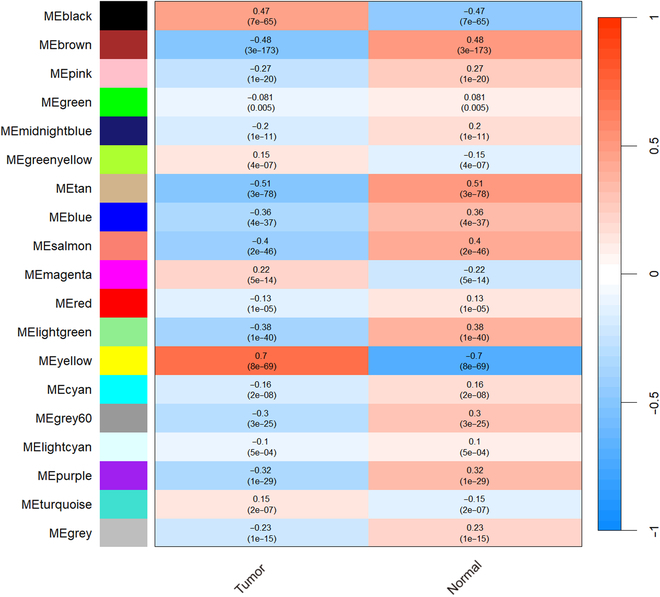
Association of modules with sample phenotypes.

For constructing specific GRNs associated with breast cancer, GO enrichment was used to analyze the enrichment pathway information of the gene module of interest. In this work, 3 modules, black, brown, and yellow, were selected for analysis. The GO enrichment pathway of modules is shown in Fig. 4. As shown in Fig. 4, the yellow module is mainly enriched in pathway chromosome segregation, nuclear division, and DNA replication. Lee et al. [26] showed that focal amplification in breast cancer usually arises from interchromosomal translocations that lead to the formation and breakage of bicentric chromosomal bridges. Yoshimoto et al. [27] concluded that the frequency of nuclear fission is one of the factors affecting the proliferation of malignant tumors in cancer. Meanwhile, it was found that some gene markers associated with breast cancer were also present in the yellow module, such as BRCA1 and CCNB1 [28,29]. In addition, using the clinical phenotypes of each sample, the correlation of each module with the sample phenotypes was calculated and is shown in Fig. 5. The yellow module can be found to have the highest correlation value with tumor. Therefore, the above module is considered a key module. Finally, 801 genes in the yellow module were screened for the construction of a GRN for breast cancer.

### Model evaluation

#### Model performance

##### TCGA database

**Fig. 6.**
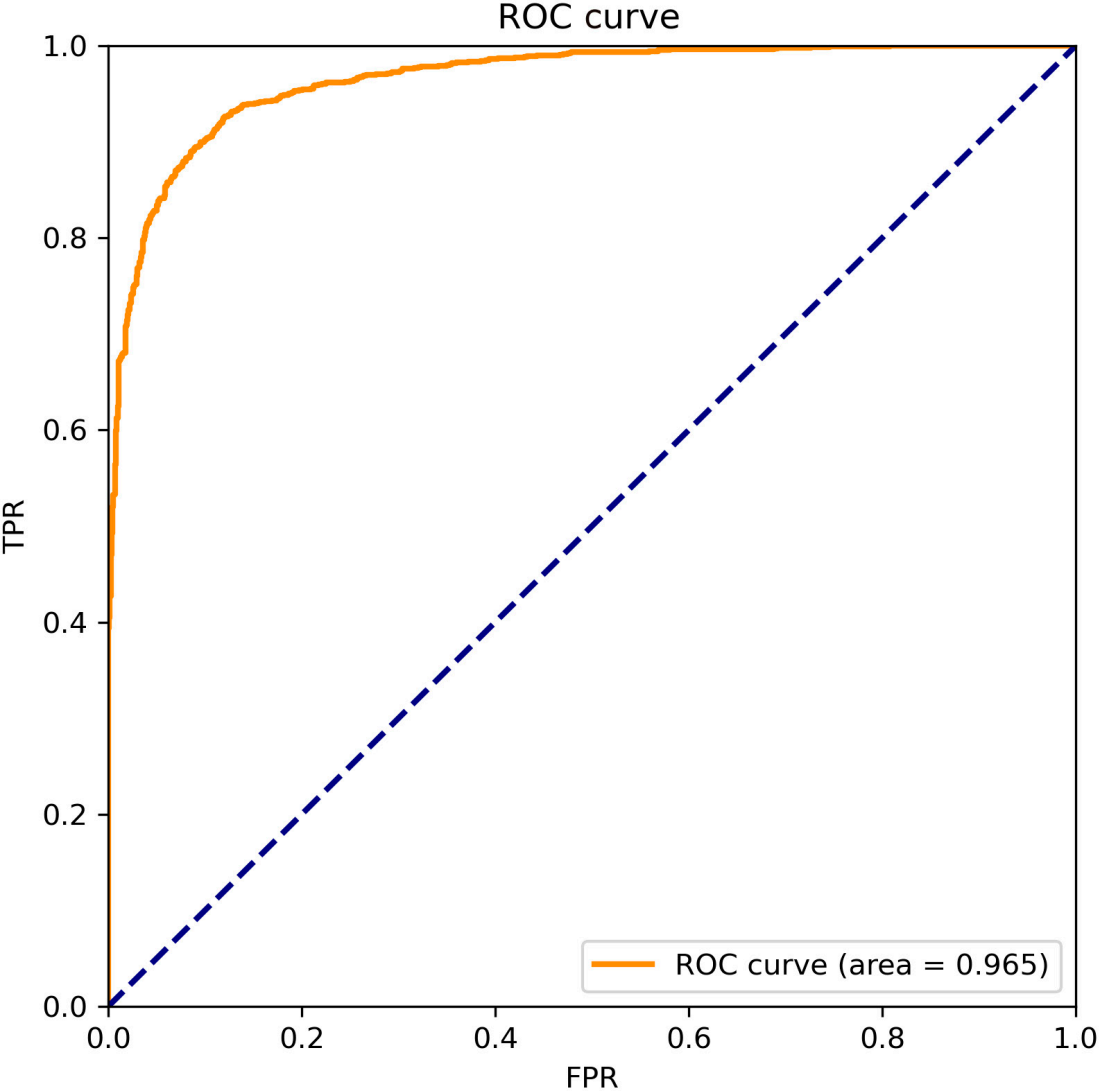
Receiver operator characteristics (ROC) curve of the model. TPR, true-positive rate; FPR, false-positive rate.

In the training stage of CNN, a 5-fold cross-validation strategy is applied to perform the forward propagation phase. During each iteration, the dataset was randomly split into 5 parts. Among them, a randomized part was used as a testing set and the rest of the dataset was used as a training set. Figure 6 exhibits the area under the curve (AUC) value achieved by the method on the testing set. The receiver operator characteristics (ROC) curve of the model is displayed in Fig. 6. It can be seen from Fig. 6 that the method has a high ability to recognize gene regulatory associations, a high sensitivity, and low misclassification rate.

**Fig. 7.**
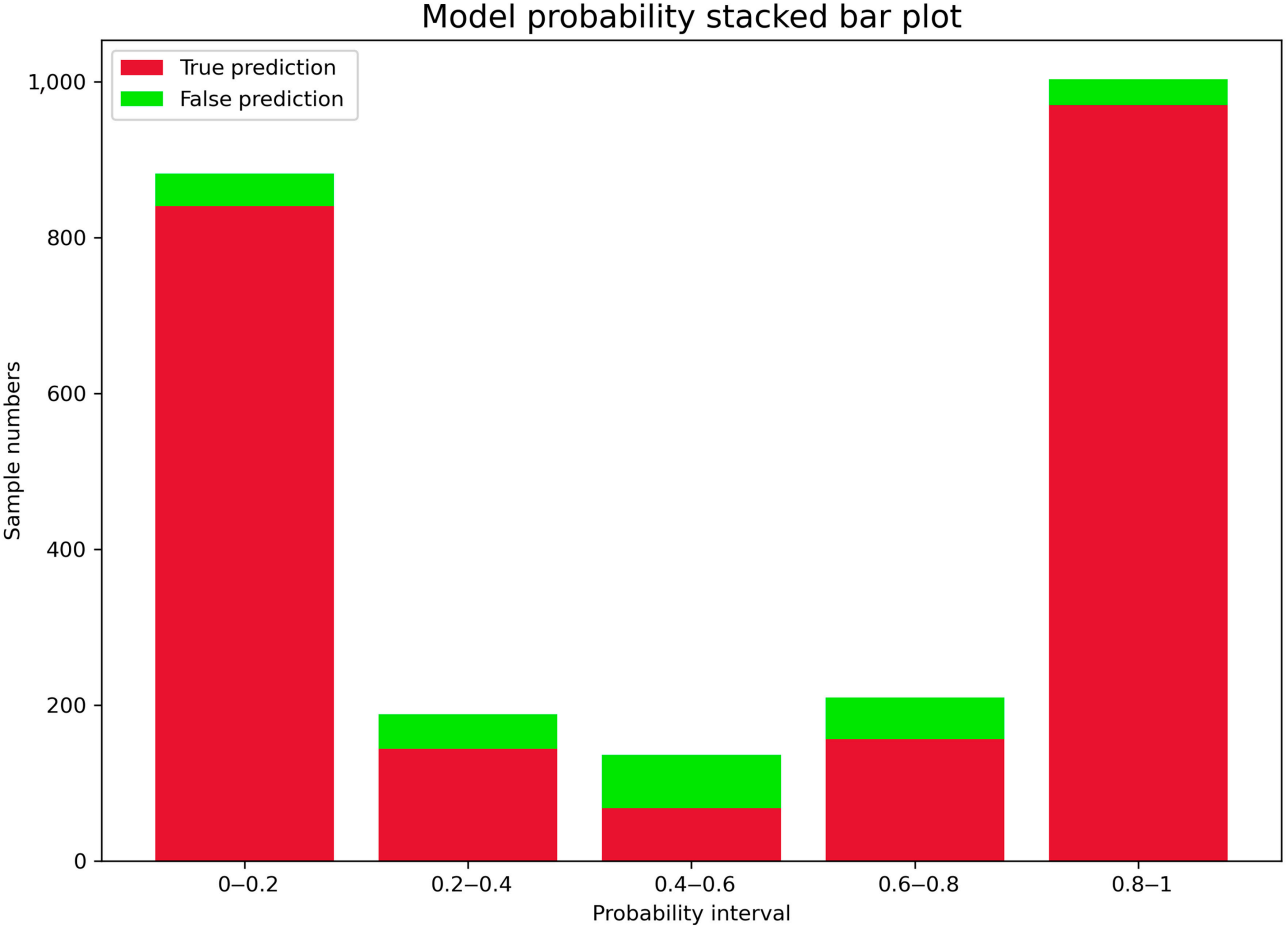
Histogram of model confidence.

The predictive accuracy of the method is assessed on the testing set. The histogram of model confidence is displayed in Fig. 7. Figure 7 reflects the probability distribution of correct and incorrect predictions of the model. It is obvious to see that the predicted probabilities of the model are mainly distributed in the ranges of [0,0.2] and [0.8,1], and the number of correctly predicted samples is markedly higher than the number of incorrectly predicted ones. It indicates that the classification results of the model have a high confidence level and are able to distinguish well whether there is a regulatory relationship between genes or not. The parameters of the relevant resource aspects of the method are as follows: total params, 549,601; trainable params, 549,601; total mult-adds, 24.34; and total size, 2.95 MB.

#### *K*-cross assessment

**Fig. 8.**
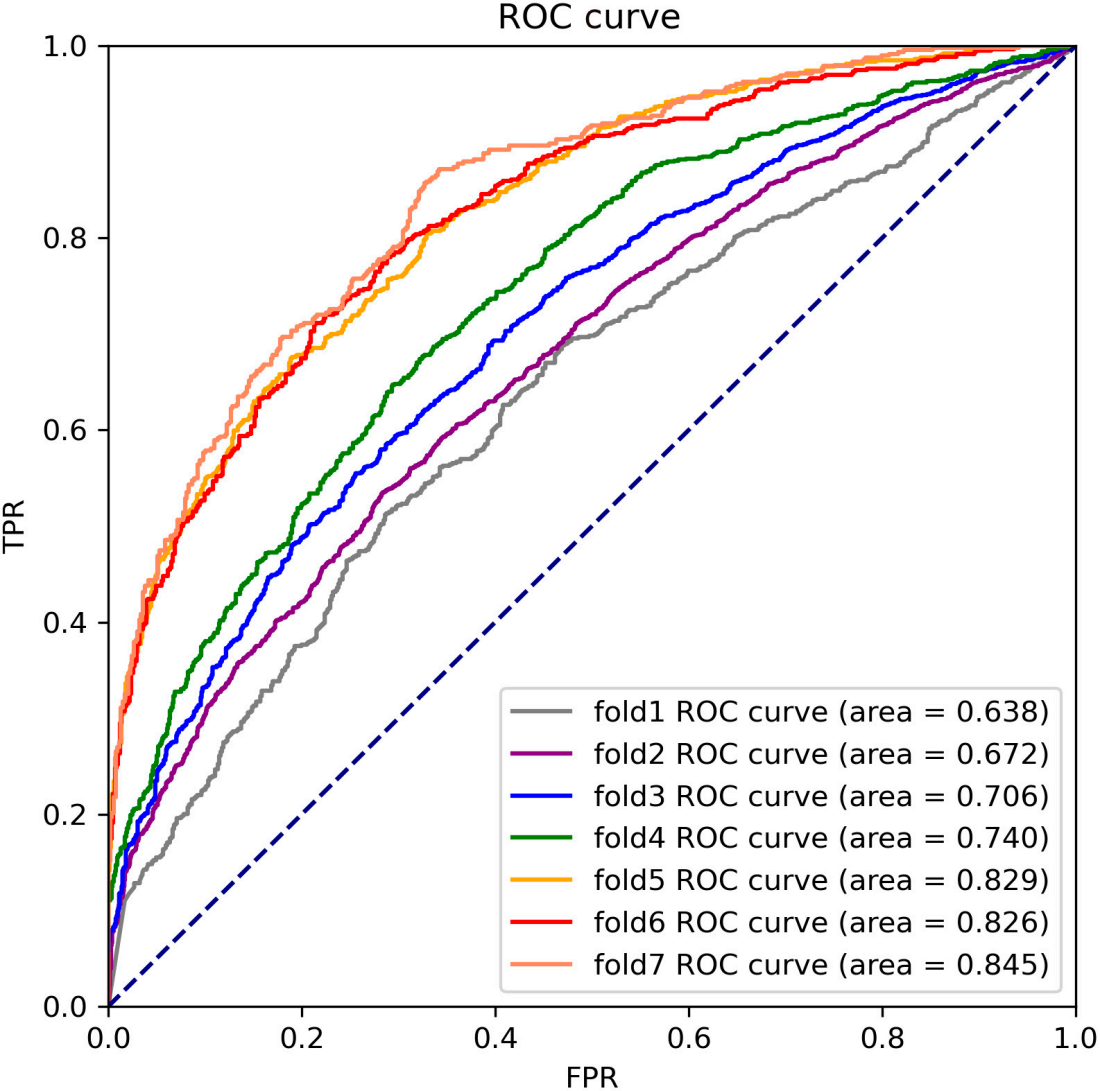
*K*-fold cross-validation.

There are 801 genes present in the screened yellow gene module. There are up to 320,400 gene associations between these 801 genes. A total of 6,157 PPI networks were retrieved using the yellow module. The PPI network retrieved using the yellow module contained 6,157 regulatory associations. The number of these regulatory associations is relatively small, so the choice was made to use cross-validation to evaluate the model. To be able to reduce the impact of cross-validation folds on the prediction results, parameter evaluation was performed. In this process, cross-validation and no cross-validation were evaluated separately. For cross-validation, the effect of the *K* values from 2 to 7 on the overall classification ability of the model was assessed. *K*-fold cross-validation is shown in Fig. 8. It shows the performance of the method on the testing set for different values of *K*. It is worth noting that for different values of *K*, the numbers of iterations on the training set are the same. After performing the evaluation, the most stable value of *K* was suggested to be 5. Therefore, 5-fold cross-validation was chosen to provide a comprehensive assessment of the method. In addition, the model training process without cross-validation was also executed during model deployment. In this process, the dataset was divided into an 8:2 ratio. The final AUC value on the testing set was 0.638. We believe that along with the accumulation of gene regulatory association information in the future, the model can get better accuracy in the separate testing set.

### Screening and validation of key genes

**Fig. 9.**
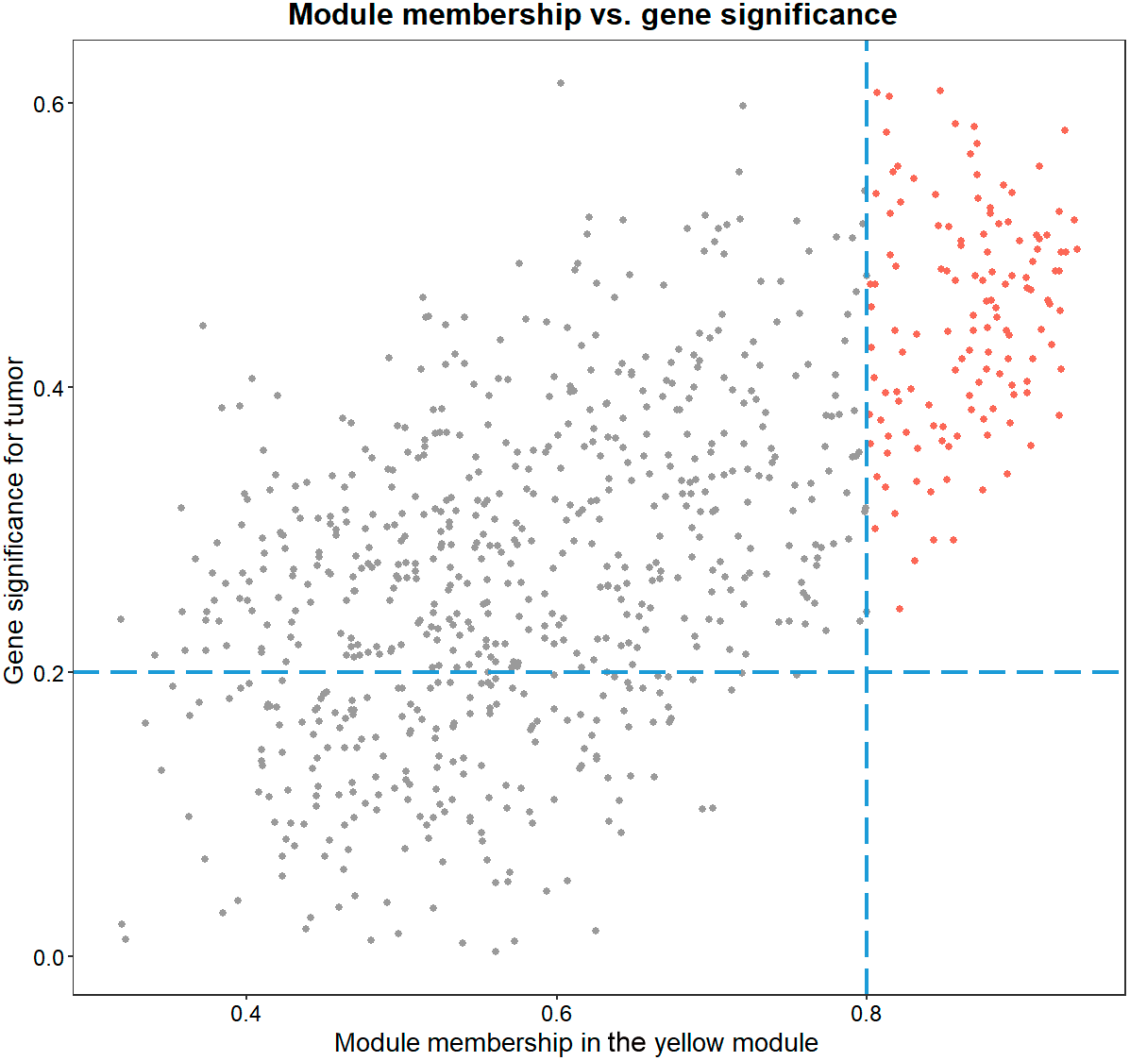
Gene significance and module membership.

Key genes play important roles in a biological process. They tend to be dominant in the pathway regulating other genes. The key genes from gene modules were screened by 2 metrics, i.e., gene significance and module membership. The gene significance and module membership of genes are shown in Fig. 9.

**Fig. 10.**
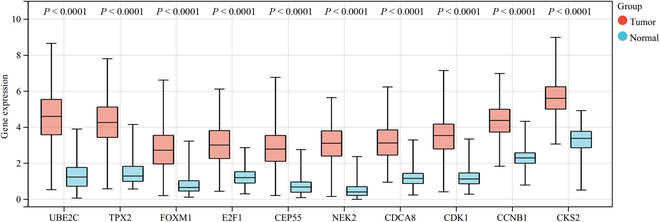
Differential expression of key genes.

**Fig. 11.**
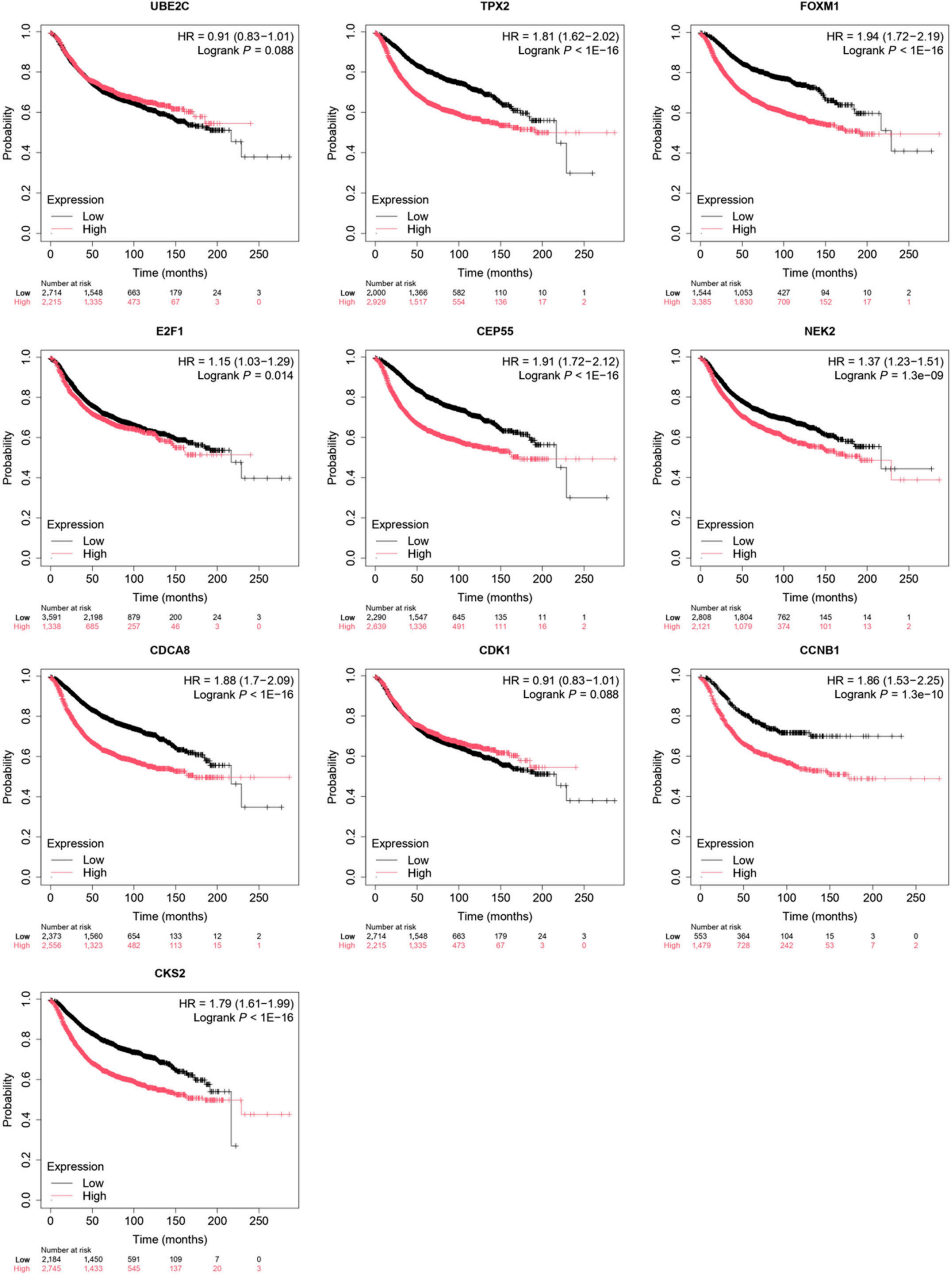
Differential expression of key genes. HR, hazard ratio.

In general, the thresholds of gene significance was set at 0.2 and the thresholds of module membership was set at 0.8 by previous research. Genes with a high gene significance ranking and module membership greater than the corresponding thresholds were considered to be key genes. The final 10 key genes screened were UBE2C, TPX2, FOXM1, E2F1, CEP55, NEK2, CDCA8, CDK1, CCNB1, and CKS2. Differential analysis provides a clear picture of gene expression and differences [30]. Differential expression of key genes is shown in Fig. 10. It shows the differential expression of key genes in the normal and tumor groups. In order to be able to further validate the correctness of the key genes, survival analysis was performed on an external sample of 4,929 patients. Survival analyses of the 10 key genes are shown in Fig. 11.

**Fig. 12.**
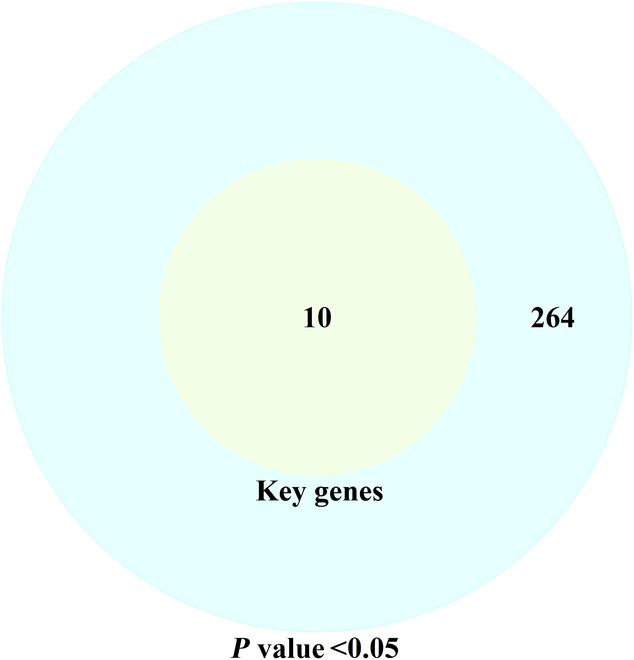
Venn diagram of key genes versus genes with *P* values <0.05.

**Fig. 13.**
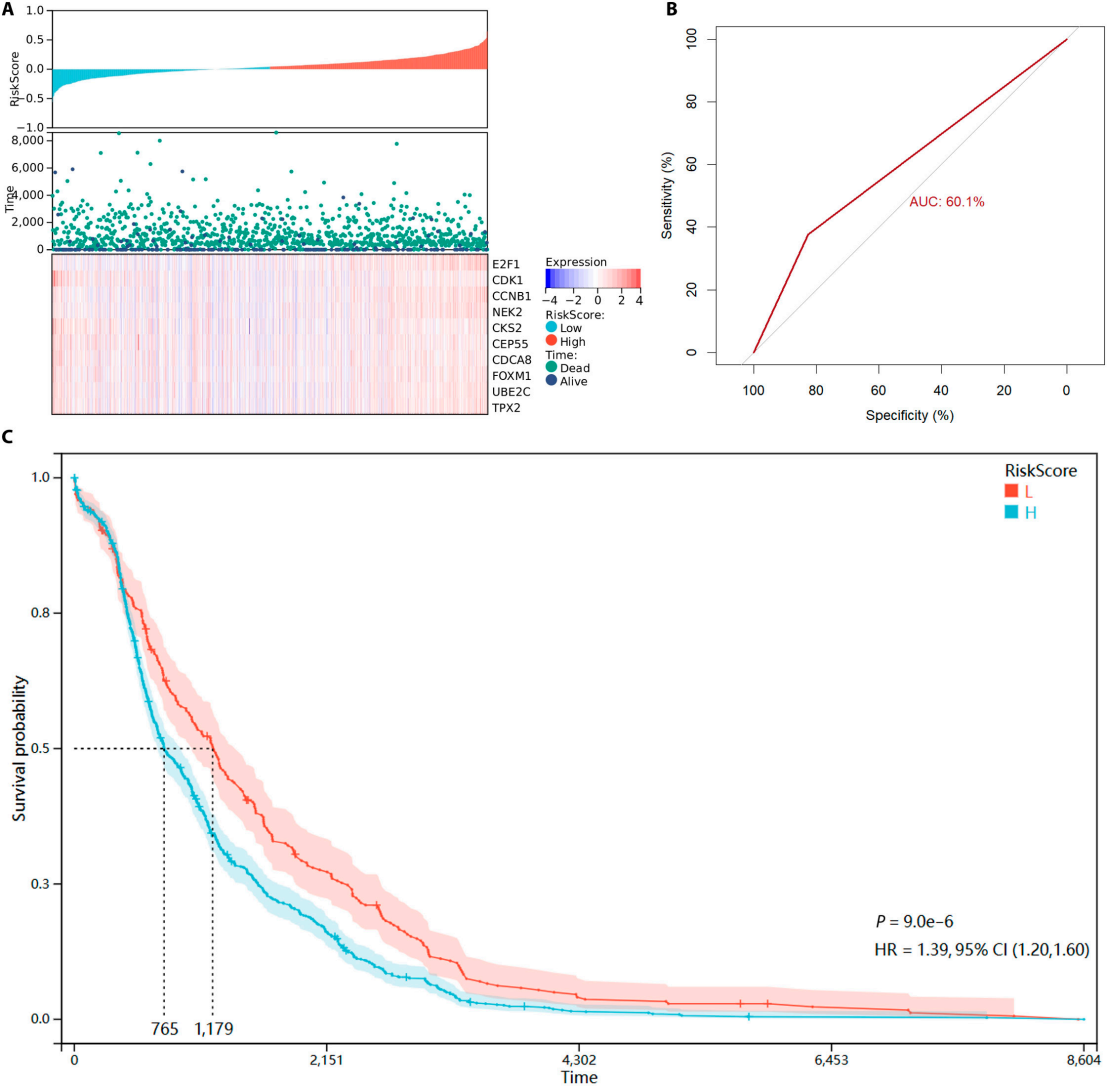
Prognostic models, ROC curves, and Kaplan–Meier (KM) curves. (A) Prognostic models for each sample based on the expression of 10 key genes. (B) Sample ROC curves under the prognostic index (PI) metric. An area under the curve (AUC) value of 0.6 confirms the validity of the PI metric. (C) KM curves based on survival times for all samples. CI, confidence interval.

The prognostic index (PI) indicator, presented in the previous section, was used to assess the importance of the selected genes. The value of PI is dependent upon the regression coefficients that are generated by the Cox regression model [31]. In the context of Cox regression, only genes with a *P* value less than 0.05 are considered to have a biological significance. The Venn diagram of key genes with *P* values <0.05 is shown in Fig. 12. It illustrates that the *P* values of the 10 key genes subjected to screening were all less than 0.05 in the Cox regression. The PI for each sample was calculated based on the regression coefficients for these 10 genes. The threshold value of PI was calculated from the median gene expression. Samples with a PI value greater than the calculated threshold were considered to be diseased, while those with a PI value below the threshold were considered to be normal. We constructed prognostic models for the samples from the expression values of key genes. In addition, ROC curves under the PI indicator and Kaplan–Meier curves were plotted. The prognostic model, ROC curves, and Kaplan–Meier curves are shown in Fig. 13.

### GRN of key genes

**Fig. 14.**
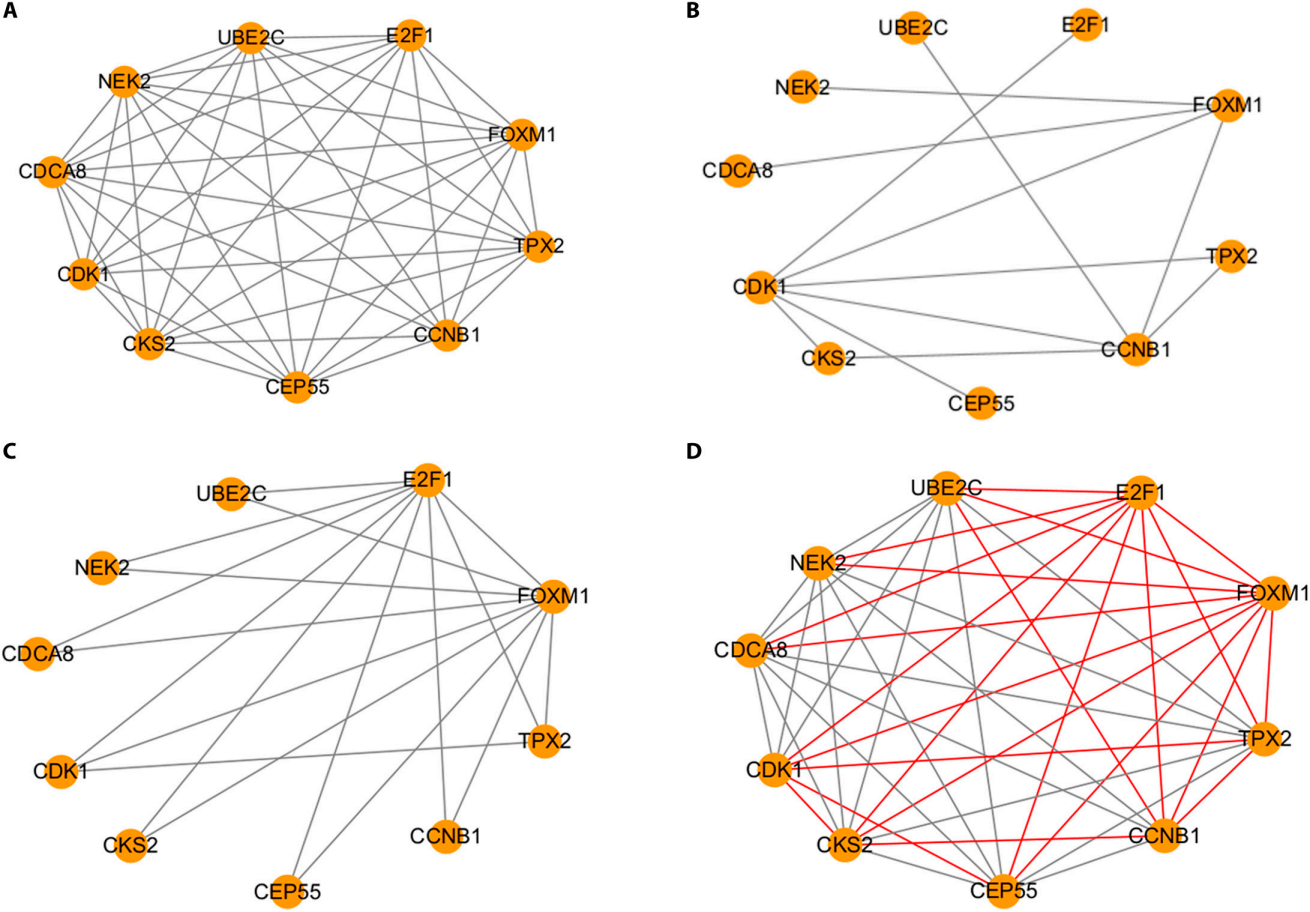
GRN of key gene regulatory association coverage. (A) The GRN of key genes was obtained based on this model. (B) Gene regulatory associations recorded in the BioGRID database. (C) Gene regulatory associations recorded in the hTFtarget database. (D) Coverage of gene regulatory associations. Red lines represent regulatory associations in the GRN that have been confirmed. Black represents that it has not been confirmed.

This section focuses on predicting regulatory associations between key genes by use of the method. In the first phase, 10 key genes were screened and obtained. Any 2 genes in key genes can be selected for transformation into a 2-dimensional (2D) histogram. Then, the association score for each key gene pair was obtained through this model. In the final classification, 0.9 was chosen as the threshold for the association score. Associations with scores above 0.9 were considered valid. As a result, 44 regulatory associations were predicted in key genes. In order to verify the accuracy of the predicted results, 2 regulatory association databases, BioGRID and hTFtarget, were selected to confirm the authenticity of the regulatory associations. BIOGRID and hTFtarget primarily record, collate, and organize data including protein, genetic, and chemical interactions covering humans and all major model organisms. The GRN of key genes, gene regulatory associations in the 2 databases, and the coverage of gene regulatory associations are visualized in Fig. 14. The results indicate that 24 regulatory pairs have been confirmed among the predicted 44 regulatory pairs.

Regulatory associations between key genes and other genes were also predicted. In this work, 2 genes were selected to perform case studies with biological experiment data, i.e., FOXM1 and E2F1. FOXM1 is an oncogenic transcription factor involved in a variety of cellular processes such as cell cycle progression, proliferation, differentiation, migration, metabolism, and DNA damage response. It is overexpressed in many human cancers, especially in breast cancer. For FOXM1, the top 15 genes with high regulatory association scores are listed in Table 1. Higher scores represent stronger regulatory association with FOXM1. It suggests that predictions of regulatory associations for FOXM1 are largely confirmed, and a precision of 93% was achieved in the top 15 scoring predictions.

**Table 1.** The top 15 genes with high regulatory association scores for FOXM1

Regulatory gene of FOXM1	Validated by hTFtarget
KIF20A	ChIP-seq evidence, epigenomic evidence, motif evidence
KIF2C	ChIP-seq evidence, epigenomic evidence
UBE2C	ChIP-seq evidence, epigenomic evidence
TPX2	ChIP-seq evidence, epigenomic evidence
SPC24	ChIP-seq evidence, epigenomic evidence
NCAPG	Unconfirmed
CDT1	ChIP-seq evidence
NEK2	ChIP-seq evidence, epigenomic evidence
HJURP	ChIP-seq evidence, epigenomic evidence
CENPF	ChIP-seq evidence, epigenomic evidence, motif evidence
CDC45	ChIP-seq evidence
CDCA3	ChIP-seq evidence
SPC25	ChIP-seq evidence
CDK1	ChIP-seq evidence, epigenomic evidence
KIF4A	ChIP-seq evidence, epigenomic evidence

ChIP-seq, chromatin immunoprecipitation sequencing

E2F1 is a key transcription factor involved in proliferation, differentiation, and apoptosis. The protein encoded by this gene is a member of the E2F family of transcription factors, which plays an important role in the control of the cell cycle and oncogene function, as well as a target of small DNA oncogenic viral transforming proteins. Previous studies have shown that the expression level of E2F1 can play a decisive role in the prognosis of breast cancer patients. For E2F1, the top 15 genes with high regulatory association scores are listed in Table 2. It shows that the top 15 scoring entries were widely confirmed in the prediction of regulatory association for E2F1. In summary, it is shown that the method is a useful tool that achieves good accuracy on real datasets and has excellent generalization capabilities. Meanwhile, the results obtained by this method are based on characteristic transformations of gene regulatory associations. A higher accuracy represents the fact that the characteristic transformations of gene regulatory associations are able to portray the relationships between genes well.

**Table 2.** The top 15 genes with high regulatory association scores for E2F1

Regulatory gene of FOXM1	Validated by hTFtarget
MYBL2	ChIP-seq evidence, epigenomic evidence, motif evidence
CDC25C	ChIP-seq evidence, epigenomic evidence, motif evidence
KIF2C	ChIP-seq evidence, epigenomic evidence, motif evidence
SKA3	ChIP-seq evidence, epigenomic evidence
DLGAP5	ChIP-seq evidence, epigenomic evidence
KIFC1	ChIP-seq evidence, epigenomic evidence, motif evidence
PLK1	ChIP-seq evidence, epigenomic evidence
NEK2	ChIP-seq evidence, epigenomic evidence
CDKN3	ChIP-seq evidence, epigenomic evidence
CKS2	ChIP-seq evidence, epigenomic evidence
MAD2L1	ChIP-seq evidence, epigenomic evidence, motif evidence
TPX2	ChIP-seq evidence, epigenomic evidence
NCAPG	ChIP-seq evidence, epigenomic evidence
NEIL3	ChIP-seq evidence, epigenomic evidence, motif evidence
RRM2	ChIP-seq evidence, epigenomic evidence, motif evidence

### Experimental verification

**Fig. 15.**
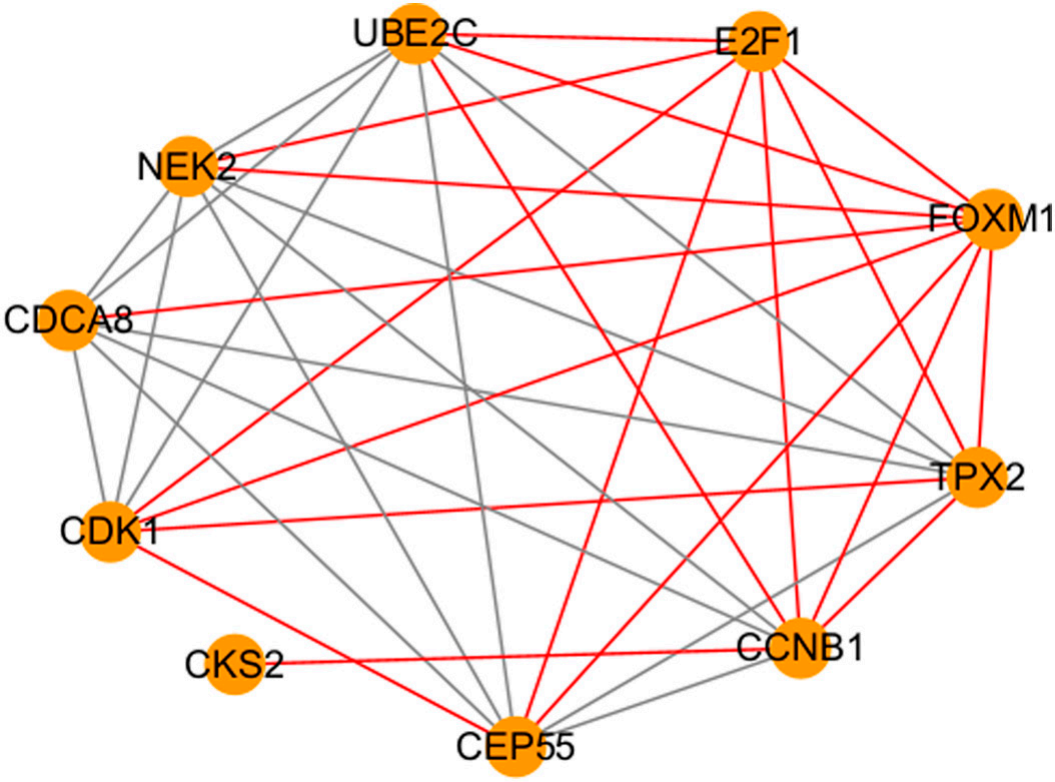
GRN of key genes predicted by random forest.

To be able to further validate the effectiveness of the method, the present method was also compared with other GRN inference algorithms. Prior to the execution of the other GRN inference algorithms, the key gene modules and the prior network were handled in the same way as in the present method. Random forest is one of the common machine learning algorithms and a common GRN inference algorithm [17]. With the same dataset, the random forest model was deployed to fit the gene regulation type. Also, GRNs between key genes were predicted by the trained model. It is worth noting that the input data for random forests are not of the histogram type. Gene regulatory associations are not characterized using histograms. For a gene regulatory association, the expression vectors of 2 genes are spliced into a longer vector of more features. Positive input samples are gene regulatory associations present in the a priori network and are labeled as 1. Negative input samples are generated by a randomized strategy and are labeled 0. Each regulatory association corresponds to a long vector and a label. The random forest model learns the type of gene regulation based on a long vector of inputs. Finally, GRNs between key genes are also predicted by the trained model. The GRN predicted by the random forest is shown in Fig. 15. After comparison with the 2 databases, it was found that 19 gene regulatory associations in GRNs were authentically recorded.

**Fig. 16.**
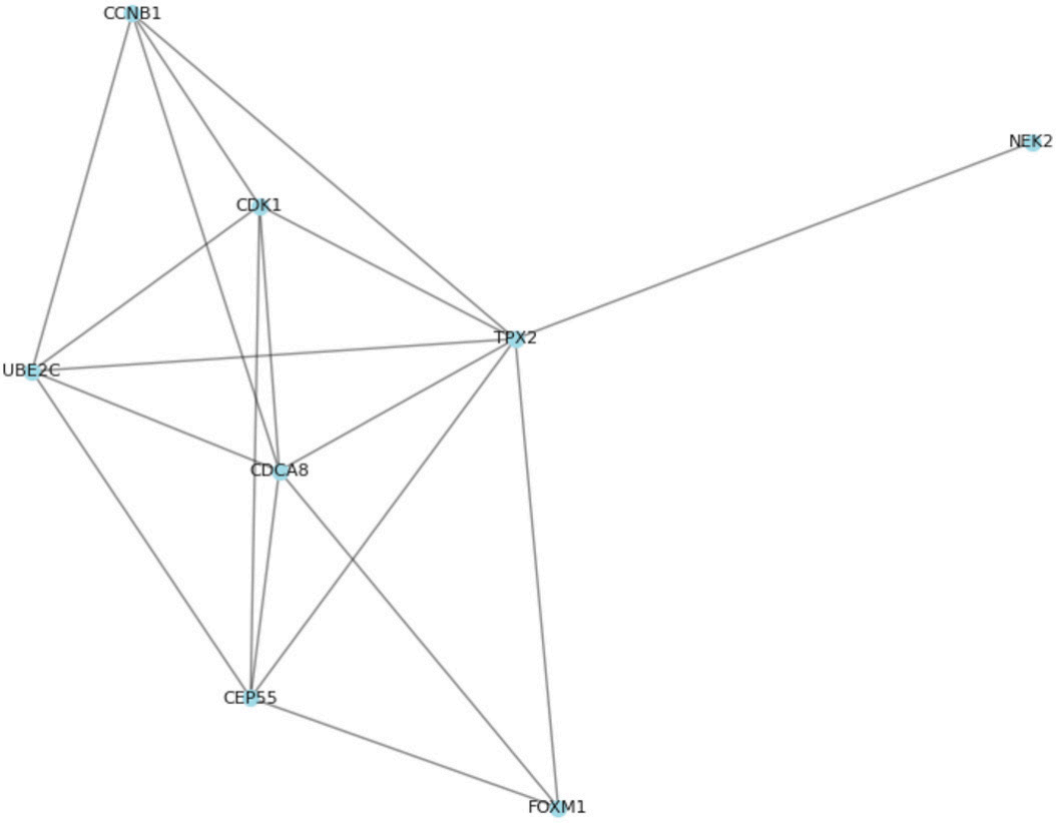
GRN of key genes predicted by mutual information.

In addition, the mutual information approach was also implemented to predict the GRN. Mutual information is a common GRN inference algorithm [3]. In this method, nonlinear relationships between gene pairs are quantified. These nonlinear relationships are calculated from the gene expression data. Prior to the execution of the method, key gene modules and key genes were acquired. Multiple nonduplicated gene pairs are generated using a list of key genes. The mutual information model then calculates a score for each gene regulatory pair based on the gene expression data. Finally, a score threshold is set to filter the predicted GRN. This threshold is obtained by calculating the quantile of all scores. The GRN between key genes was predicted by the mutual information model and is displayed in Fig. 16. After comparison with the 2 databases, it was found that 8 gene regulatory associations in the GRN were authentically recorded. The comparison results of the 3 models are also displayed in Table 3. It can be found that among the 3 models, the CNN achieves a higher AUC and predicts more of the proven regulatory edges in the GRN.

**Table 3.** Comparison of the 3 models

Model	AUC	Number of edges in GRN	Proven edges of gene regulation
WGCNA + CNN	0.965	44	24
WGCNA + random forest	0.806	35	19
WGCNA + mutual information	0.562	18	8

WGCNA, weighted gene coexpression network analysis

Further, the generalizability of the model was verified. The dataset at Gene Expression Omnibus (GEO) database number GSE25066 was chosen as the input data for the method. WGCNA and enrichment analyses were performed on this dataset to screen for key genes and key gene modules. The final module selected on this dataset was the brown module. Although the color of the module was different from the one selected in the TCGA dataset, 9 of the key genes screened from the brown module were the same as the key genes in the yellow module on the TCGA database. It is worth stating that the colors of the modules in WGCNA are related to the input data and may be different for different data. In this dataset, the key genes selected in the brown module have a high degree of overlap. This can indicate that the division of gene modules is reliable. Then, the brown module was utilized to obtain the PPI network as a priori network. The CNN was used as the main framework to fit the gene regulation type and predict the GRNs between key genes. Finally, the AUC value of the method on this dataset was 0.97. The GRN between the 9 key genes was also predicted. There are 31 regulatory edges in this GRN, and 15 were confirmed. This shows that the method has some prediction accuracy in the GSE25066 dataset as well.

## Conclusion

In the present study, a novel method combining genetic module and deep learning is proposed to infer the specific GRNs of breast cancer. From the perspective of gene coexpression networks, gene regulatory associations are investigated in our method. This method is a strategy for analyzing gene expression data, and it also combines deep learning to fit and explain the regulatory relationships between genes. In our method, WGCNA and GO enrichment analysis were performed to screen for key genetic modules. Meanwhile, the key genes in the key gene module were screened and validated from multiple angles. The results of differential analysis, survival analysis, and prognostic analysis fully demonstrate the accuracy of inferred key genes. The PPI in the key genetic module was selected as the a prior network. In the PPI network, each gene regulatory association from the prior network is transformed into a 2D histogram representation. Each gene regulatory association corresponds to a histogram and a label. Then, CNNs are fitted as the main framework to predict regulatory relationships among key genes. The AUC and confidence level on the testing set reflect the predictive accuracy of the model. Meanwhile, the results of comparison with multiple methods show that the method can infer more proven gene regulatory associations. In addition, the GRNs of key genes were inferred by this model. Many of the regulatory associations in GRNs are extensively validated in 2 publicly available databases and have high coverage. In addition, more detailed analysis was carried out for 2 key genes. Among the top 15 gene regulatory associations, the exact presence of gene regulatory associations covered up to 90%. The method was shown to have good accuracy in predicting gene regulatory associations.

## Materials and Methods

### Data processing and data collection

The RNA-seq data of breast cancer was downloaded from TCGA database and represents the gene expression value. The dataset contains 1,217 samples and 19,938 genes. The level of gene expression in this dataset is measured by FPKM (fragments per kilobase of transcript per million mapped reads). The data consist of multiple sample files, and each file contains expression values for all genes. RStudio (http://www.r-project.org/) is an integrated development environment for the R programming software, which is a software platform in the bioanalytical field [32]. It was applied to integrate these files and record them in matrix *I*. The matrix *I* is called an expression matrix and defined as

$$I=\left[\begin{array}{ccc} I_{11} & \cdots & I_{1n}\\ \vdots & \ddots & \vdots \\ I_{m1} & \cdots & I_{\mathrm{mn}} \end{array}\right]$$ (1)

where *m* refers to the total number of genes and *n* refers to the total number of samples. $I_{\mathrm{ij}}$ represents the expression value of *i*th gene in *j*th sample. Since the expression values of individual genes are relatively large and carry large weights on the results, logarithmic transformation was used here to reduce the differences in expression between genes [33]. Therefore, $I_{\mathrm{ij}}$ can be reassigned as

$$I_{\mathrm{ij}}=\text{log}\left(1+I_{\mathrm{ij}}\right)$$ (2)

During the growth and development of organisms, the expression of genes is affected by many factors, and it may be very different even in the same process. In statistics, median absolute deviation (MAD) is a robust measure of the sample deviation for univariate numerical data [34]. It is a criterion used to characterize univariate samples that are variable in quantitative data. MAD has better robustness compared to standard deviation. The standard deviation is easily affected by individual large deviations in the data, whereas for MAD, a small number of outliers will not affect the final result. In the biomics data, genes can be eliminated by the MAD criterion and it can be described as

$${\mathrm{mad}}_{i}=\text{median}\left(I_{\mathrm{ij}}-\text{median}\left(I_{i}\right)\right)$$ (3)

where $\text{median}\left(I_{i}\right)$ represents the median expression of *i*th gene in all samples. Considering that genes with large expression differences have a crucial impact on the experimental outcome, a suitable threshold value was set. If the absolute values of genes were greater than the threshold value, these genes would be regarded as abnormal expression genes; otherwise, they were retained.

Further, outlier samples need to be eliminated to make the experimental results more precise. Based on the newly obtained matrix *I*, we applied the Euclidean distance method to measure the distance between samples [35]. The *i*th column of the matrix *I* is selected as the vector *S*(*i*). *S*(*i*) represents the expression value of all genes in the *i*th sample that are numeric vectors whose entries mirror the expression profile of all genes. Then, the distance between sample *S*(*i*) and sample *S*(*j*) is calculated by

$$\text{Dist}\left(S\left(i\right),S\left(j\right)\right)={\left\|\left(S\left(i\right)-S\left(j\right)\right)\right\|}_{2}$$ (4)

Therefore, the distance between all the samples can be obtained by Eq. (4). It indicates the gap between the *i*th sample and the *j*th sample. Threshold α is set to filter outlier samples. Samples with a distance exceeding the threshold are eliminated; otherwise, they will be retained. After the processing of data and the elimination of samples, we obtained the expression values of 15,000 genes in 1,173 samples.

### Genetic module screening

Generally, construction of GRNs about a certain disease has an important meaning for disease treatment. However, organisms contain thousands of genes during growth and development, which is not conducive to the construction of specific GRNs. Therefore, gene coexpression patterns were analyzed to extract genes most associated with the specified disease. In this subsection, coexpression patterns between genes were analyzed by WGCNA aiming to screen genetic modules significantly associated with tumor phenotypes. Moreover, the key genes in the genetic modules were analyzed to construct specific GRNs. WGCNA is an efficient system biology approach to describe correlation patterns between different samples [36,37]. This approach aims to seek coexpressed genetic modules, explore the associative relationships between gene networks, and excavate the key genes. In our work, WGCNA has 4 important processes, namely, selecting a soft threshold, constructing gene coexpression networks, identifying modules, and screening modules.

#### Selection of a soft threshold

In our work, package of R software was used to implement WGCNA of the specified disease and extract important genes. The expression matrix *I* was obtained in the previous stage as input for the construction of the WGCNA network. In general, the strength of relationships between genes is measured by correlation coefficients such as Pearson correlation [38]. It is defined as follows:

covxy=covxyσxσy=EX−EXY−EYσxσy=EXY−EXEYEX2−E2XEY2−E2Y (5)

where $\mathrm{cor}\left(x,y\right)$ is the covariance between the numerical vectors *x* and *y*. $\sigma_{x}$ and $\sigma_{y}$ represent the variances of vector *x* and *y*, respectively. Different genes play different roles in the growth and development of organisms. A few genes are extremely important, but more genes have little effect. Only using the strength of correlation between genes is not enough to portray their expression patterns. Scale-free networks have good robustness: they will not produce a large change in the network structure if a node in the network is removed [39]. In WGCNA, the correlation between genes is defined as follows:

$$a_{\mathrm{ij}}={\left|\frac{1+\mathrm{cor}\left(X_{i},X_{j}\right)}{2}\right|}^{\beta }$$ (6)

where $X_{i}$ is the expression value of the *i*th gene in all samples and it is the *i*th row in the expression matrix *I*. $\mathrm{cor}\left(X_{i},X_{j}\right)$ is calculated by using the Pearson correlation coefficient. The correlation between all genes can be calculated by Eq. (6), and matrix $A=\left[a_{\mathrm{ij}}\right]$ is a correlation matrix to record the degree of correlation between genes. β is the value of soft-thresholding power. In order to make the gene coexpression network conform to a scale-free network distribution, β must be strictly set. If β is set to a fixed value (hard threshold), it is not possible to accurately portray gene-to-gene coexpression, which can result in the loss of information from the real network. In the present study, β was selected from 1 to 20 to evaluate the distribution of gene expression. Finally, the most suitable soft threshold was obtained based on the input matrix *I*.

#### Construction of a coexpression network

After selecting the most suitable soft threshold, weighted correlations between genes were recorded in adjacency matrix *A* by Eq. (6). In this network, each different gene acts as a separate node and the edge is the gene-to-gene connectivity strength. Larger values of $a_{i}j$ in *A* represent a stronger association between 2 genes, and vice versa. However, the relation between 2 genes is not a direct effect of the 2 genes but is also indirectly affected by other genes. Considering that neighboring genes have direct and indirect effects on gene expression, topological overlap was used to measure complex correlations between genes. It is defined as follows:

$${\mathrm{TOM}}_{\mathrm{ij}}=\frac{\sum_{u\neq i,j}A_{\mathrm{iu}}A_{\mathrm{uj}}+A_{\mathrm{ij}}}{\min\left(K_{i},K_{j}\right)+1-A_{\mathrm{ij}}}$$ (7)

$$K_{i}=\sum_{u}A_{\mathrm{iu}}$$ (8)

where $K_{i}$ denotes the sum of the linkage strengths of the *i*th gene to all other genes. The *TOM* matrix is obtained based on the correlation matrix *A*, which is more capable of portraying the true relationship between genes.

#### Identification and filtering of genetic modules

Genetic modules are highly interconnected sets of genes. The division of gene modules facilitates the analysis of large numbers of genes, such as functional enrichment, trait association analysis, and sample association analysis. In order to identify genetic modules in the present study, hierarchical clustering was applied to discriminate the module category of each gene [40]. Each genetic module and sample phenotype have different levels of correlation strength. It is important to find the modules that are most relevant to the tumor being explored in constructing GRNs. For the purpose of narrowing down genes and building targeted GRNs, the following 2 aspects were used as the main reference points: (a) GO enrichment was used to analyze the pathways enriched by individual modules and select the modules associated with tumor pathogenesis [41]. (b) Search for current tumor gene markers, and observe which module they belong to. Since genetic modules are collections of genes, each inline module is summarized and classified by its first principal component gene as the module’s feature, also known as module eigengene. Further, using the module eigengene, the correlation of the gene modules with the clinical phenotypes of the samples was calculated for screening the most relevant modules. Therefore, the above points were used to select the gene modules most relevant to the tumor.

#### Selection of key genes

In order to be able to predict regulatory associations between genes more accurately, the screened modules were subjected to specific treatments to access key genes. The key genes were screened primarily through gene significance and module membership. Gene significance refers to the degree of association of a gene with sample trait, and module membership means the association strength of gene expression profiles and gene modules. The module membership was set to a reasonable threshold of 0.8 to filter out some of the genes. Then, genes are ranked by gene significance, and the ranking represents the importance of the gene. The genes were also validated by a variety of metrics, mainly including difference analysis and survival analysis [42,43]. Here, PI was used to measure the correctness of key gene screening:

$${\mathrm{PI}}_{j}=\sum_{i}\beta_{i}*I_{\mathrm{ij}}$$ (9)

where ${\mathrm{PI}}_{j}$ represents the value of the *j*th sample, $\beta_{i}$ is the Cox regression coefficient of the *i*th gene, and $I_{\mathrm{ij}}$ is the expression value of the *i*th gene in the *j*th sample. The PI value for each sample will be obtained. Then, the threshold for PI is calculated from the median expression of key genes. If the PI value of a sample is greater than this threshold, a label of diseased is attached, and vice versa, a label of normal is attached. In this way, a prognostic indicator was constructed to measure the accuracy of genetic screening.

### Gene regulatory association prediction

In this subsection, the regulatory associations between genes in the gene module were transformed into 2D histograms. Meanwhile, a CNN was used to fit association types and predict GRNs between key genes. This was inspired by Chereda, who used a graph CNN to predict the patient-specific molecular subnetworks of breast cancer metastasis with the Human Protein Reference Database PPI network as the prior knowledge [44,45]. To infer specific GRNs that are significantly related to the tumors being explored, PPI is employed to construct GRNs. The PPI network is retrieved from Search Tool for Recurring Instances of Neighbouring Genes (STRING) as the prior network. The STRING database (https://string-db.org/) is a searchable database of protein interactions [46]. The database can be applied to 2,031 species and contains 9.6 million proteins and 13.8 million protein interactions. It contains experimental data, results mined from PubMed abstracts, and synthesized data from other databases, as well as predicted results using bioinformatics methods [47].

After obtaining the key gene module associated with the tumor, the collection of genes in the module is selected as a list. The PPIs in this gene collection are retrieved from STRING. Consequently, the exact associations contained in the modules screened in the previous stage are extracted. It is worth noting that in our experiments, we only investigated whether there was a regulatory relationship between genes and did not make a specific distinction between positive and negative regulations. In PPI, there are regulatory associations between genes and these gene regulatory associations are also known as positive samples. These positive samples are labeled 1. In this work, negative samples are generated by a randomized strategy. Firstly, the set of genes in the key gene module is selected as a list. Second, one gene in the list is randomly selected as endpoint 1 of the negative sample. Another gene is randomly selected as endpoint 2 of the negative sample. If 2 genes have already been recorded in the PPI, this generation is skipped. Otherwise, the negative sample is generated and the label is recorded as 0.

#### Feature conversion

In order to be able to tap into the GRNs embedded in gene sets, a CNN was chosen as the main framework. A gene regulatory association corresponds to 2 genes, and each gene corresponds to an expression vector. In order to be able to integrate the association information embedded in the expression vectors of the 2 genes, gene regulatory associations are transformed into 2D histogram types. The specific conversion method is as follows: Here, it is assumed that there is a regulatory relationship between gene *a* and gene *b*. Firstly, the maximum and minimum expression values of gene *a* are obtained in all samples. These 2 values are equivalent to the maximum and minimum elements of the row vector in which gene *a* is located in the expression matrix *I*, denoted as $\max_{a}$ and $\min_{a}$, respectively. Similarly, the maximum and minimum values of gene *b* are $\max_{b}$ and $\min_{b}$, respectively. Secondly, the interval $\left[\min_{a},\max_{a}\right]$ is equalized into 32 subintervals for gene *a*. Similarly, the interval $\left[\min_{b},\max_{b}\right]$ is equally divided into 32 subintervals for gene *b*. Thirdly, we create the 32 ∗ 32 matrix *H*. Then, the 32 small ranges of gene *a* serve as the horizontal scale of matrix *H*, and the 32 small ranges of gene *b* serve as the vertical scale of matrix *H*, or vice versa. Finally, from the perspective of the expression value of gene *a* and gene *b*, each sample is assigned to matrix *H*. Consequently, each entry of *H* records the number of samples that satisfy the horizontal and vertical scales. Matrix *H* acts as the 2D histogram that indicates the association of gene *a* and gene *b*.

#### Structure of the model

The classification decision-making power of CNNs is used to learn the GRNs embedded in gene expression data. This task is depicted as a binary classification of gene-to-gene association 2D histogram $H_{32*32}$ to a target variable $Y\in \left\{0,1\right\}$. In the present study, the main idea is to transform gene-to-gene regulatory association into a 2D histogram based on gene expression data. It is important to note that GRNs are an undirected graph $G=\left(V,E,D\right)$, where *V* and *E* record the sets of vertices (genes) and edges (gene-to-gene association), respectively, and *D* stands for the adjacency matrix of the graph. After transforming all gene-to-gene association edges into a 2D histogram, the input data of the model can be represented as a binary group $\left(H,Y\right)$, where *H* represents the 2D histogram corresponding to the 2 endpoint genes of the link edges and *Y* represents the corresponding labels. The overall mapping function of the model is as follows:

$$Y=\varphi \left(H\right)$$ (10)

where *H* is the input image and *Y* is the output of the input image obtained from the mapping function. In particular, the mapping function φ contains several convolutional layers, pooling layers, and fully connected layers. A convolutional kernel of size 3∗3 was used to sense the information contained in the localized regions of the input image. Each convolution kernel is obtained as follows:

$$C{\left(x\right)}_{i,\;j}^{k}=\sum_{m=0}^{2}\sum_{n=1}^{3}w_{i+m,\;j+n}^{k}X_{i+m,\;j+n}^{k}$$ (11)

where *X* is the input image of the current network layer, which is the output of the previous network layer. *i* and *j* are the coordinates representing where the output neuron is located. *w* is a convolutional kernel whose size can be set flexibly. In our study, the size of all convolutional kernels is 3 ∗ 3. Wang et al. [48] min factorized convolutional networks and evaluated the contribution of convolutional kernels of sizes 3 ∗ 3 and 5 ∗ 5 to the model. The results showed that the 5 ∗ 5 convolutional kernel has little performance improvement for the model, and the 3 ∗ 3 convolutional kernel has fewer parameters and is more flexible. *k* is the index number of the convolutional kernel.

Due to the forward propagation of the input data through the model, which is costly and consuming, we applied several 2 ∗ 2 maxpooling layers for down sampling in our experiments. The role of a maxpooling layer is to remove redundant information, compress features, simplify network complexity, reduce computation, reduce memory consumption, achieve nonlinearity, and expand the perceptual field. The function for the maxpooling layer is defined as

$$\text{maxpooling}{\left(X\right)}_{i,\;j}=\max\left(X_{2i,\;2j},\;X_{2i+1,2j},\;X_{2i,\;2j+1},\;X_{2i+1,\;2j+1}\right)$$ (12)

where *X* is the output coordinate and $\left(i,j\right)$ determine the position of the output elements. In fact, the elements of the current layer take the largest value within a specific size range of the previous layer. In addition, the linear activation function *Relu* is used to increase the nonlinear fitting ability of the neural network. Since the last layer of our network is binary classification, the probability is calculated using the *Sigmoid* function for the last layer. The functions *Relu* and *Sigmoid* are defined as

$$\text{Relu}\left(x\right)=\left\{\begin{array}{ll} 0 & x<0\\ x & x\geq 0 \end{array}\right.$$ (13)

$${\text{Sigmoid}}_{\theta }\left(x\right)=\frac{1}{1+e^{\theta x}}$$ (14)

In particular, overfitting is a problem that must be considered during the use of CNN models. In our study, several dropout layers were introduced to improve the generalization ability and robustness of the model. The main role of the dropout layer is to randomly and temporarily discard a portion of neurons with a probability *P*. Specifically, due to its mechanism of randomly discarding some neurons, a neural network is trained with a different structure in each iteration. The dropout layer function is defined as

$$r=\mathrm{rand}\left(\right)$$ (15)

$$\text{dropout}\left(x\right)=\left\{\begin{array}{cc} 0 & P\\ \frac{x}{1-P} & 1-P \end{array}\right.$$ (16)

where *x* is the input value of the current neuron and *P* is a predetermined probability threshold set to 0.25. This formula represents that neurons will be zeroed out with probability *P*, while surviving neurons are upscaled by a factor of 1/(1 − *P*).

#### Strategy of training and evaluation

In this work, there were several strategies, as follows: (a) The training dataset was divided into positive and negative samples, where the negative samples were generated by a randomized strategy. (b) A 5-fold cross-validation strategy was applied to train the model. To be specific, during each iteration, the data were equally divided into 5 subgroups. One of the groups served alone as the testing set, and the remaining 4 groups were used as the training set. There are multiple pairs of gene regulatory associations between genes, but the number of gene associations recorded in the current a priori network is low. Therefore, cross-validation was chosen to evaluate the model. Additionally, the false-positive rate (FPR) and true-positive rate (TPR) were calculated at different thresholds. Based on the computation of FPR and TPR, the ROC curve was drawn to evaluate the classification ability of the model. Similarly, the 2D histogram is 2-dimensional, where the rows represent gene *a*, the columns represent gene *b*, and each element represents the number of samples in which gene *a* and gene *b* are in a specific expression range. It is worth noting that the 2D histogram is employed as input of the model and is labeled just 0 or 1. The reason for this is that this study aims at mining the regulatory associations between genes and does not discuss the types of regulatory relationships. A study on the type of regulation will be our future work.

The main idea of this method for inferring GRNs is to transform the inference problem into a classification problem. In order to evaluate the performance of the method, several common metrics are used and defined as follows:

$$\text{Accuracy}=\frac{\mathrm{TP}+\mathrm{TN}}{\mathrm{TP}+\mathrm{TN}+\mathrm{FP}+\mathrm{FN}}$$ (17)

$$\mathrm{TPR}=\frac{\mathrm{TP}}{\mathrm{TP}+\mathrm{FN}}$$ (18)

$$\mathrm{FPR}=\frac{\mathrm{FP}}{\mathrm{FP}+\mathrm{TN}}$$ (19)

where *TP*, *FP*, *TN*, and *FN* stand for the numbers of correctly predicted regulatory pairs (true positive), incorrectly predicted regulatory pairs (false positive), correctly predicted nonregulatory pairs (true negative), and incorrectly predicted nonregulatory pairs (false negative), respectively.

#### Model generalizability

To further validate the generalizability of the model to other datasets, dataset number GSE25066 in the GEO database was chosen as the input data for the method. The GSE25066 dataset contains expression values for 12,548 genes in 508 samples. The preprocessing process for this dataset was consistent with the TCGA data processing section, and key gene modules were also screened for analysis via WGCNA and GO enrichment. Similarly, the process of transforming the features of gene regulatory associations is consistent with the main framework of the model.

## Data Availability

The results and source code related to this study are available at https://github.com/Chenyihao109/WGCNA_CNN.git.
